# Synthesis and Characterization of 2-Decenoic Acid Modified Chitosan for Infection Prevention and Tissue Engineering

**DOI:** 10.3390/md19100556

**Published:** 2021-09-29

**Authors:** Carlos Montez Wells, Emily Carol Coleman, Rabeta Yeasmin, Zoe Lynn Harrison, Mallesh Kurakula, Daniel L. Baker, Joel David Bumgardner, Tomoko Fujiwara, Jessica Amber Jennings

**Affiliations:** 1 Department of Biomedical Engineering, The University of Memphis, Memphis, TN 38152, USA; cwells3@memphis.edu (C.M.W.); cclman22@memphis.edu (E.C.C.); ryeasmin@memphis.edu (R.Y.); zlhrrson@memphis.edu (Z.L.H.); mkrakula@memphis.edu (M.K.); jbmgrdnr@memphis.edu (J.D.B.); 2 Department of Chemistry, The University of Memphis, Memphis, TN 38152, USA; dlbaker@memphis.edu (D.L.B.); tfjiwara@memphis.edu (T.F.)

**Keywords:** chitosan, biomaterials, electrospun, acylation, antimicrobial, local delivery

## Abstract

Chitosan nanofiber membranes are recognized as functional antimicrobial materials, as they can effectively provide a barrier that guides tissue growth and supports healing. Methods to stabilize nanofibers in aqueous solutions include acylation with fatty acids. Modification with fatty acids that also have antimicrobial and biofilm-resistant properties may be particularly beneficial in tissue regeneration applications. This study investigated the ability to customize the fatty acid attachment by acyl chlorides to include antimicrobial 2-decenoic acid. Synthesis of 2-decenoyl chloride was followed by acylation of electrospun chitosan membranes in pyridine. Physicochemical properties were characterized through scanning electron microscopy, FTIR, contact angle, and thermogravimetric analysis. The ability of membranes to resist biofilm formation by *S. aureus* and *P. aeruginosa* was evaluated by direct inoculation. Cytocompatibility was evaluated by adding membranes to cultures of NIH3T3 fibroblast cells. Acylation with chlorides stabilized nanofibers in aqueous media without significant swelling of fibers and increased hydrophobicity of the membranes. Acyl-modified membranes reduced both *S.* *aureus* and *P.* *aeruginosa* bacterial biofilm formation on membrane while also supporting fibroblast growth. Acylated chitosan membranes may be useful as wound dressings, guided regeneration scaffolds, local drug delivery, or filtration.

## 1. Introduction

Chitosan is considered a promising therapeutic delivery agent due to its biodegradability, biocompatibility, non-toxicity, and inherent antimicrobial activity [1,2]. Chitosan is a sugar-based biopolymer derived from exoskeletons of arthropods, e.g., crustaceans and insects, fungi cell walls, mollusks radulae, fish scales, cephalopod beaks, and lissamphibian skin. Structurally, chitosan is a heteropolymer composed of *N*-acetyl-d-glucosamine and d-glucosamine unit connected through β (1-4) glycosidic bond. Chitosan has three reactive functional groups: an amine group at the C-2 position, and primary and secondary hydroxyl groups at C-6 and C-3 positions, respectively. Chitosan is polycationic at a pH below six and interacts with negatively charged molecules, such as proteins, anionic polysaccharides, fatty acids, bile acids, and phospholipids [3]. Chitosan is a versatile polymer due to its flexibility that allows manufacturing into various forms such as gels, nanofibers, pastes, films, etc. Electrospun chitosan membranes are of particular interest for biomedical applications due to their porous nanofibers and high surface area that mimics the extracellular matrix. Multiple biomedical applications, including wound dressings, drug delivery, and tissue engineering, involve nanofibrous chitosan membranes [4,5].

Chemical modification of electrospun chitosan membranes can enhance their physicochemical properties, further functionalizing the material to allow for a broader range of applications. For example, the incorporation of hydrophobic substituents, such as fatty acids, generates a domain for absorbing and carrying poorly soluble drugs. Literature supports the potential for fatty acid (FA)-treated chitosan membranes to control the release of the hydrophobic drug simvastatin [6]. Linoleic and α-linolenic acid-modified chitosan has demonstrated potential as a multifunctional catheter coating by improving the lubricity and antimicrobial properties [7]. A study also found that fatty acid incorporated chitosan can improve mucoadhesive properties in a self-nano-emulsifying drug delivery system [8]. Studies investigated decanoic acid grafted chitosan as a potential carrier of insulin by combining the mucoadhesive and permeative properties of chitosan and decanoic acid, respectively [9]. Decanoic, oleic, and linoleic acid-modified chitosan have enhanced wound healing rates [10,11]. The length of the fatty acyl chain incorporated through O-acylation can control the chitosan nanofiber’s crystal structure. It also improves its stability in the moist environment while maintaining its non-toxic property and has shown promise for regenerating bone in guided bone regeneration (GBR) applications in rodent models [12,13,14]. A study using buriti oil containing volatile compounds and fatty acids indicated that chitosan and buriti oil could be combined into a gel to improve chemical properties and activity against Gram-negative pathogens [15]. In addition to the antimicrobial activity, chitosan gel with buriti showed antioxidant and anti-inflammatory properties, good healing activity, and an adequate wound retraction rate [15].

Trifluoroacetic acid (TFA) is one of the most commonly used solvents for electrospinning chitosan membranes because it provides adequate viscosity for the polymer solution to be pulled into nanofibers [6,16]. Despite this benefit, TFA forms a salt with chitosan’s amino groups, requiring removal without compromising the nanofibrous structure or deteriorating the membrane’s mechanical properties. One technique to achieve this balance involves grafting fatty acid (FA) groups to the hydroxyl groups outside of the chitosan fibers to create a hydrophobic covering to prevent fiber swelling during the washing steps of TFA ions [13]. FA chains can be attached to any of the three reactive groups; acid chlorides and methanol crosslinks FAs in the amine position [17,18]. Acylation reactions may also use a coupling agent, such as 1-ethyl-3-(3-dimethyl-aminopropyl)-1-carbodiimide hydrochloride (EDC) to improve the reactivity [7]. The TFA salt in the electrospun chitosan membrane occupies the amine group [16]. Wu et al. developed an O-acylation method in which the chitosan membrane is acylated by acid anhydride in the presence of a pyridine catalyst to improve its stability in an aqueous solution [12,14].

The fatty acid 2-decenoic acid (2DA) and its analogs are medium chain FA chemical messengers naturally produced by bacteria. Studies have shown that the *cis* form of 2DA (C2DA) disperses existing biofilm and inhibits biofilm formation [19]. Studies suggest that 2DA could increase microbes’ metabolic activity and the bactericidal ability of commonly used antimicrobials [20]. These properties could make 2DA a potential complementary therapy for infection. Additionally, 2DA could lessen antibiotic tolerance by improving the efficacy of these drugs against biofilm infection. Acylating chitosan membranes with 2DA or analogs may provide the advantages of bacterial biofilm resistive materials and the ability to load with hydrophobic therapeutics for extended release. However, 2-decenoyl chloride (2DC) is not commercially available. This study investigates a custom synthesis route for acyl chlorides and their ability to stabilize and functionalize chitosan nanofibers. Additionally, this study determined physicochemical properties, antimicrobial properties, and cytocompatibility of acyl-modified chitosan nanofibers [21].

## 2. Results

### 2.1. Viscosity Average Molecular Weight

The intrinsic viscosity (η) of the chitosan used for electrospinning membranes was determined to be 6.249. The calculated viscosity average MW was 664.7 kDa.

### 2.2. Fabrication

**Figure 1:**
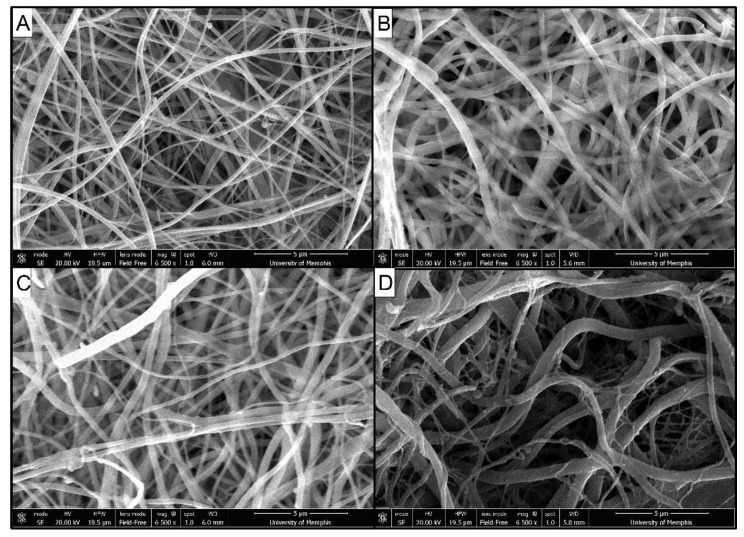
Scanning electron microscope images of (**A**) as-spun, (**B**) decanoic treated, (**C**) hexanoic treated, and (**D**) 2-decenoic treated membranes at 6500× magnification.

**Figure 2:**
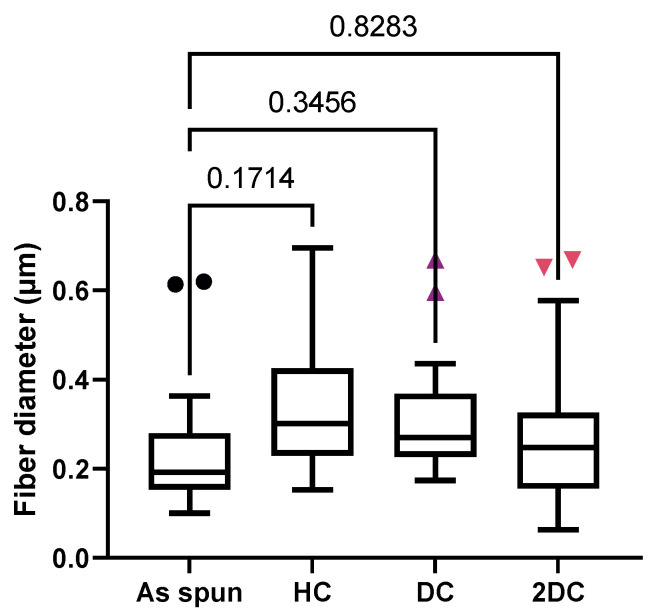
Boxplot shows fiber diameters after acylation. Numbers above connected lines are *p*-values for comparisons. Outliers are shown as individual points.

Scanning electron microscope images showed that fibers formed and stabilized by each acylation method without significant swelling (Figure 1). No significant differences in fiber diameter were detected between treated groups and as-spun membranes (Figure 2).

### 2.3. Fourier Transform Infrared (FTIR)

**Figure 3:**
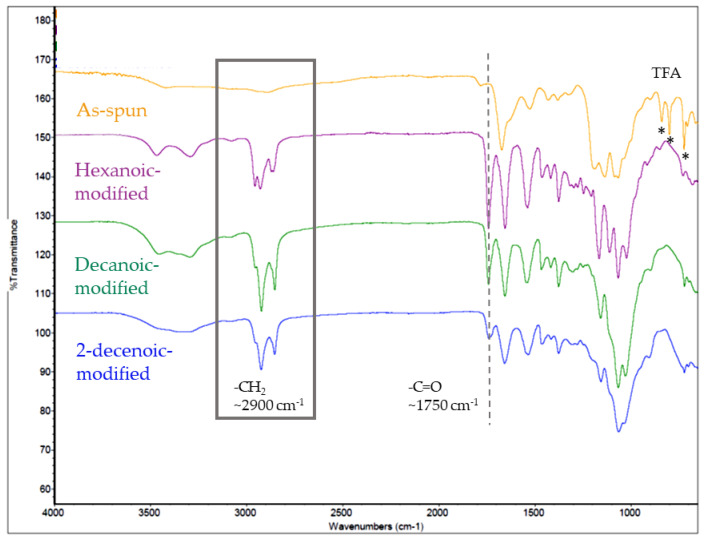
FTIR spectra of chloride modified and as-spun nanofibrous chitosan membranes. Transmittance values have been offset in to facilitate interpretation.

Sharp peaks at 1750 cm^−1^ were observed in FTIR spectra for HC, DC, and 2DC modified membranes (Figure 3). Peaks around 2900 cm^−1^ also confirm acyl carbon chains at the surface of the treated membranes, with increased intensity with increasing FA chain lengths. The two peaks around 3300 and 3500 cm^−1^ for DC modified and HC modified membranes represent NH2. The lack of peaks < 1000 cm^−1^ in treated membranes confirms the removal of TFA salts [14,22].

### 2.4. Contact Angle

**Figure 4:**
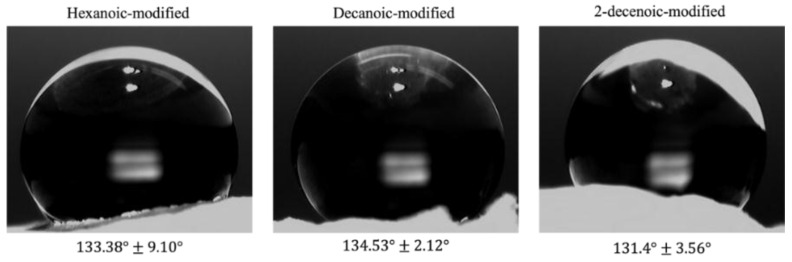
Picture of water droplet on modified chitosan membranes. Values are mean ± standard deviation (*n* = 3).

Water droplets remained stable on modified membranes for more than 5 min (Figure 4). Among all the treatments, decanoic-modified membranes were the most hydrophobic (134.53° ± 2.12°). Contact angle measurements on as-spun membranes were not possible due to dissolution of the chitosan-TFA salt upon placement of the drop.

### 2.5. Thermogravimetric Analysis (TGA)

**Figure 5:**
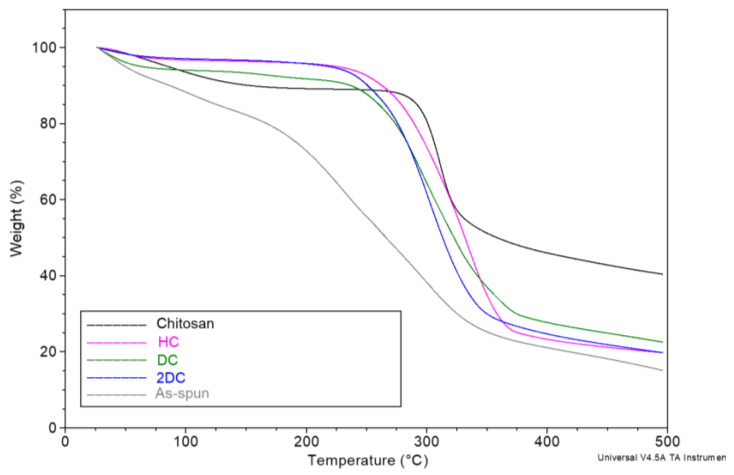
TGA (10 °C min^−1^, raw data) of chitosan (86.5% DDA), HC, DC, 2DC modified and as-spun chitosan membranes.

Original chitosan powder (86.5% DDA) lost approximately 10% of its mass initially, which is characteristic for chitosan due to water bound by strong hydrogen bonding with hydroxyl (-OH) groups. Acyl-modified membranes lost approximately 2–5% of total mass initially, as less water was bound to hydrophobic membranes (Figure 5). Non-modified chitosan had an initiation (T_D_ (I)) (°C) temperature of onset of 263.3 °C, where acyl modified membranes had (T_D_ (I)) (°C) values of 215.43 °C, 216.6 °C, and 212.34 °C for hexanoic-, decanoic-, and 2-decenoic- modified membranes, respectively (Table 1).

**Figure 6:**
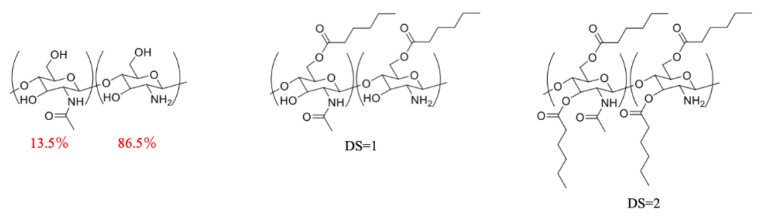
Representation of 86.5% deacetylated chitosan units before surface modification with hexanoyl chloride, center) surface modified chitosan with a degree of substitution (DS) = 1, and right) surface modified chitosan with a DS = 2.

Chitosan is composed of a glucosamine unit (C_6_H_13_NO_5_) with a molecular weight (MW) of 179 and a N-acetyl glucosamine unit (C_8_H_15_NO_6_) with a MW of 221. The chitosan used during this process is 86.5% deacetylated (Figure 6, left). Equation (1) represents the average monomer MW.

Average chitosan MW (86.5% DDA) = 179 × 0.865 + 221 × 0.135 = 184.7(1)

The surface modification of chitosan with the various modifiers does not affect the monomer backbone of chitosan. This allows the theoretical calculation for degree of substitution (DS) per chitosan unit, which is structurally represented in Figure 6 (center and right).

From Table 1, at 500 °C chitosan has degraded by 55%, which resulted in 45% remaining. Using results from Equation (1) and the following calculations, the average of remaining unit MW can be determined.

184.7 × 0.55 = 101.6, degraded(2)

184.7 × 0.45 = 83.1, remaining(3)

Since fatty acids evaporate with cleavage of the ester linkage at the stage of Deg-1, it can be assumed that the same amount (weight) remains at 500 °C in the original chitosan and modified chitosan membranes. The following calculations estimate the DS per chitosan unit for each surface modifier. HC (C_6_H_11_O) has a MW of 98, DC (C_10_H_19_O) has a MW of 154, and 2DC (C_10_H_17_O) had a MW of 152. Equation (4) estimates DS for HC, Equation (5) estimates DS for DC, and Equation (6) estimates DS for 2DC.

(184.7 + 98*x*) × 0.21 = 83.1, *x* = 2.1 (4)

(184.7 + 154*x*) × 0.25 = 83.1, *x* = 1.0 (5)

(184.7 + 152*x*) × 0.21 = 83.1, *x* = 1.4 (6)

### 2.6. Antimicrobial Activity

**Figure 7:**
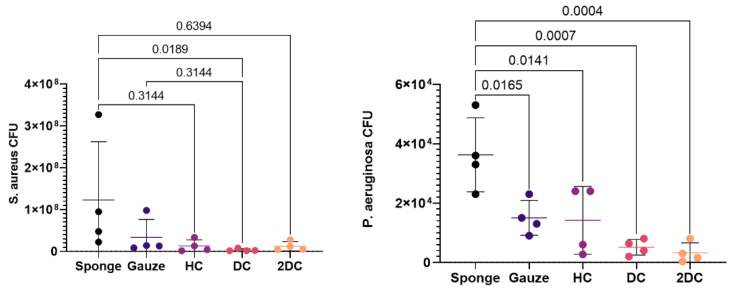
Scatterplot shows colony forming units (individual points shown with lines representing mean ± standard deviation) of *S. aureus* (left) and *P. aeruginosa* (right) for the treated membranes were compared with sponge and gauze controls. Numbers above connected lines are *p*-values between groups.

The sponge control had significantly more CFUs counted than all other groups (Figure 7). The CFU count for hexanoic treated membranes was comparable to the CFU count for the gauze control; however, the CFU counts for decanoic treated and 2-decenoic treated membranes was significantly less than the gauze control.

**Figure 8:**
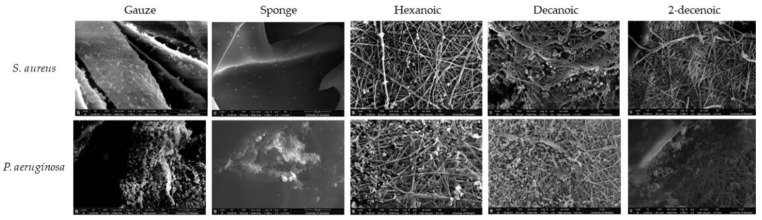
SEM images of biofilms attached to the gauze, sponge, and acylated membranes.

SEM images of biofilm attached to membranes confirmed that some sparse colonies of *S.* *aureus* exist on hexanoic- and decanoic-acylated membranes, with very few observed on 2-decenoic membranes. *P.* *aeruginosa* formed abundant EPS on gauze fibers (Figure 8). In contrast, while *P.* *aeruginosa* subsisted on acylated membranes, EPS formation was minimal.

### 2.7. Cytocompatibility

**Figure 9:**
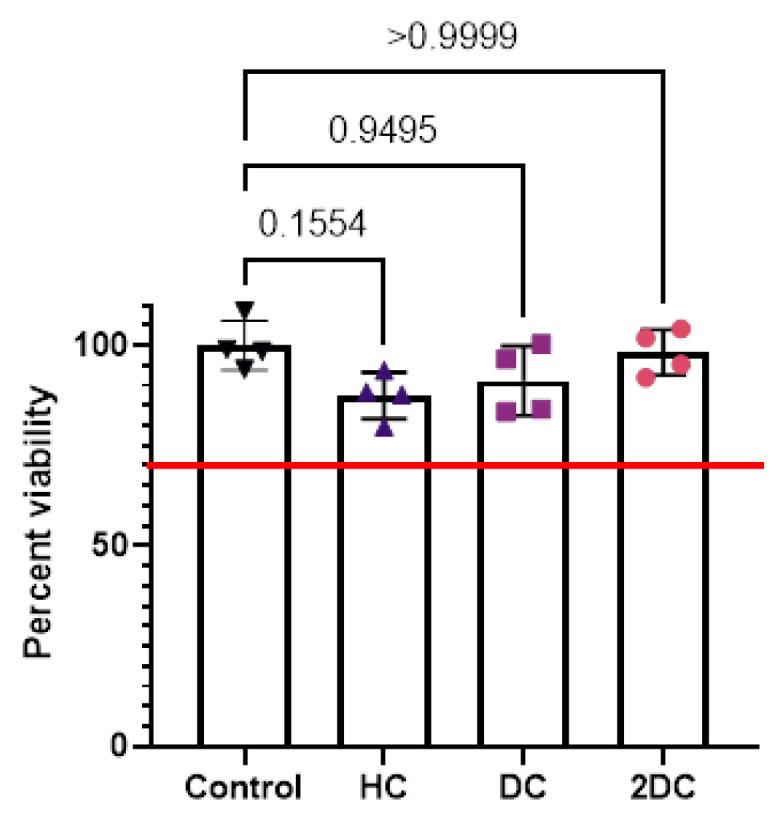
Graph shows cytocompatibility testing of acyl modified membranes in transwell in contact with NIH 3T3 cells (*n* = 4). Individual data points are shown as symbols with bars representing mean and error bars representing standard deviation. Red line indicates the 70% cytocompatibility minimum as established by ISO 10993-5.

The percent viability of cells exposed to modified membranes showed no significant differences, and all were above the 70% cytotoxicity threshold, in accordance with the ISO 10993-5 Biological Evaluations of Medical Devices standard when evaluating biomaterials against fibroblasts [23] (Figure 9).

## 3. Discussion

The study results demonstrate successful synthesis of the acyl chloride of 2-decenoic acid and modification of nanofibers without affecting fiber diameter. Synthesized chlorides are customizable, making previously commercially unavailable compounds accessible for acylation processes. Synthesized chlorides can modify various chitosan-based biomaterial properties in a nondetrimental way, particularly in that the functionalization imparts hydrophobic properties that limit bacterial attachment and may also allow loading and release of therapeutics [6,7]. The ability to synthesize chlorides that are not commercially available could expand the possible applications to other fatty acid analogs, including *cis*-2-decenoic acid and 2-heptylcyclopropane-1-carboxylic acid [24,25], to expand possible antimicrobial solutions in the continuing fight against antibiotic resistance and complex biofilm-associated infections.

FTIR results indicate immobilization of FAs on the fibers. The absorption peak around 1750 cm^−1^ representing the acyl group (C = O) and ester bond formation confirms acylation. Ester bonds may be particularly advantageous for these materials in infection prevention. In the presence of acidic environments, such as those found locally at tissue injury sites or in the presence of bacterial enzymes, such as lipase, they may hydrolyze [19,20]. Environment-influenced hydrolysis may cause acylated chitosan biomaterials to be less reactive until interaction with bacteria or damaged tissue. This study did not measure the hydrolysis rate of fatty acids; future studies will investigate whether conjugated 2DA release is lipase or pH-sensitive. FTIR spectra broad peaks at 3100–3500 cm^−1^ represent inter- and intra-molecular hydrogen bonding of the -NH2 and -OH vibration stretching of chitosan molecules [13]. Of note, TFA-salt representative transmittance peaks at 720, 802, and 837 cm^−1^ are not present in any of the modified chitosan biomaterials that confirm the salts are no longer present.

Water contact angle measurements also validate the acylation process that imparts hydrophobic properties to the hydrophilic chitosan biomaterial. The contact angle results for this study using acyl chlorides are consistent with prior studies that used acyl anhydrides [6,12] in that the contact angle increases with the chain length. Although contact angle on as-spun membranes was not measured, contact angles for acylated membranes are higher than those observed for neutralized chitosan films and coatings (80–100°) in other studies [26,27]. Decanoic acid and 2-decenoic acid have the same chain length, with 2DA having one unsaturated bond. The unsaturated fatty acid should have less hydrophobicity than the saturated decanoic acid, which is consistent with the lower contact angle for 2DA-modified membranes. The variability in contact angle observed in this evaluation may be due to varying degrees of substitution, as well as the rough texture of the nanofibers. A further limitation is that membrane surfaces were not completely flat, which could introduce error or variability to the measurements.

Chitosan is known to thermally degrade in two phases under nitrogen atmosphere; one phase occurs around 300 °C with complete mass loss over 600 °C [28]. As acyl chains cap the hydroxyl groups, membranes become more hydrophobic with less water associated, which explains why less mass of water is lost during the initial phase of TGA for acyl-modified membranes. The earlier initiation and onset temperatures for acyl-modified membranes are consistent with other studies of ester-modified chitosans [29,30], and provide additional confirmation that ester linkages are occurring. The larger percentage of total mass lost after the onset of degradation for acyl-modified membranes are likely due to the degradation of alkyl chains, also confirming that acylation was successful. The calculated theoretical degree of substitution for hexanoic-modified membranes is in agreement with values for similar materials obtained by elemental analysis [12]. The DS of 2 for hexanoic-modified membranes suggests that, per chitosan unit, both primary alcohol and secondary alcohol reacted with the hexanoic chloride. The lower degree of substitution for decanoic- and 2-decenoic modified may be due to steric hindrance of the additional carbons on the chains. The higher degree of substitution for 2-decenoic-modified membranes compared with decanoic-modified may be due to the trans-unsaturation point making the carbonyl more accessible for higher reactivity.

Acylated chitosan membranes demonstrated the ability to inhibit bacterial growth and attachment (CFUs). In all antimicrobial testing conditions, the acylating nanofibers showed evidence of reduced biofilm attachment. Surface attachment is a mechanism biofilm uses to develop and persist. Modified chitosan-nanofibrous membranes have more surface area for bacteria to attach than chitosan sponge or gauze and still produced better bacteria inhibition results. These findings support the hypothesis that acyl-modification contributes to improved antimicrobial properties. Acyl-modified materials seem to inhibit *P. aeruginosa* EPS production. Reduction of EPS secretion from *P. aeruginosa* blocks a primary mechanism *P. aeruginosa* uses to form a biofilm, and modified materials may interfere with type IV pili [31,32,33]. *S. aureus* biofilm inhibitory effects may be due to interference with microbial surface components recognizing adhesive matrix molecules (MSCRAMM). MSCRAMMs are instrumental in *S. aureus* attachment and subsequent biofilm formation [34]. The differences in bacterial hydrophobicity/hydrophilicity may explain the differing degrees of response between *S. aureus* and *P. aeruginosa* [35,36]. The high variability observed for CFU counts may be overcome in future studies by using different types of viability assays, such as luciferase-based luminescence assays. When unattached bacteria remain in the planktonic state longer, they are more susceptible to antimicrobials and the innate immune system. In this study *S. aureus* and *P.* aeruginosa were chosen as representative Gram-positive and Gram-negative strains that are common pathogenic strains in bone and wound injuries. More studies of efficacy of modified membranes against other bacterial and fungal strains are necessary to understand their broad antimicrobial efficacy.

Balancing bacterial inhibition with cyto- and bio-compatibility is challenging for many potential antimicrobial biomaterials, drug delivery systems, and tissue regeneration templates [37,38,39,40]. All acyl-modified materials demonstrated cytocompatibility with no detectable differences between any of the evaluated groups. All modified membranes met or exceeded the minimum 70% cellular compatibility threshold recommended by the ISO 10993-5 Biological Evaluations of Medical Devices standard [23]. Future studies will evaluate the effects of these materials on other cell types, such as immune cells, and will assess biocompatibility in vivo. While this study did not assess as-spun material as controls due to the rapid dissolution and acidity of these materials, the acyl-modified materials performed similarly to previously investigated chitosan-based materials [6,41,42,43]. There are no signs of acyl-modified materials adversely affecting cells or any signals that healing would be negatively affected [10].

In summary, modified chitosan biomaterials possess characteristics that support their use in infection prevention treatment strategies. These methods allow for functionalization of chitosan with specific fatty acids. Future studies will evaluate conjugated fatty acid hydrolysis rate in physiological relevant solutions, including acidic and in the presence of enzymes such as lipase. Additional future and ongoing studies will characterize the drug delivery capabilities of acylated nanofiber biomaterials loaded with therapeutics such as local anesthetics, statins, chemotherapeutics, or antimicrobials.

## 4. Materials and Methods

### 4.1. Characterization of Viscosity Average Molecular Weight

The average molecular weight of chitosan (Chitolytic; Toronto, ON, Canada) was validated using viscosity measurement. To determine the intrinsic viscosity (*η*), chitosan was dissolved in various concentrations (0.07, 0.08, 0.13, 0.20 g/dL) 0.25 M acetic acid and 0.25 M sodium acetate and filtered through a 0.45 μm filter. Flow time of the solvent and different concentrations of CS samples were measured at 25 ± 0.1 °C using an Ubbelohde viscometer [44]. The intrinsic viscosity and average molecular weight of CS were calculated using the following Mark–Houwink–Sakurada (MHS) equations.

Intrinsic viscosity [*η*] = *KM^a^* (7)

Viscosity average molecular weight *M* = ([*η*]/*K*)^1/*a*^ (8)

where viscometric constant *K* = 1.57 × 10^−3^ cm^3^/g and *a* = 0.79 [45,46] for a solvent of 0.25 M acetic acid and 0.25 M sodium acetate.

### 4.2. Fabrication of Electrospun Membranes

Nanofibrous chitosan membranes were electrospun using chitosan (86.5% DDA) following previous methods [43]. Briefly, Chitosan was dissolved overnight at 5.5% (*w*/*v*) in 70:30% (*v*/*v*) TFA and dichloromethane (DCM) purchased from Sigma Fisher (Burlington, MA, USA). The solution was centrifuged to remove any insoluble chitosan, transferred to a syringe with a 20-gauge blunt needle, and electrospun at a rate of 15 µL min^−1^ and a voltage of 27 kV using a syringe pump onto an aluminum foil covered collector plate rotating at ~8.4 revolutions per minute, with constant monitoring of the Taylor Cone to ensure high-quality membranes. The electrospinning apparatus was housed inside a ventilated box which was vented to a fume hood. The apparatus was operated at room temperature and at 40–60% humidity. Membranes were spun from three 10 mL volumes to obtain a diameter of 15 cm and thickness of approximately 700 µm. After membranes were fabricated, they were sectioned into 10 mm diameter discs for use in experiments.

### 4.3. Synthesis of 2-Decenoyl Chloride

A reflux reaction was used to synthesize 2-decenoyl chloride based on the method described by Namazi et al. [47], by first placing 1 M (40 g L^−1^) of sodium hydroxide (NaOH) in a covered beaker on ice. The NaOH beaker was connected to a condenser unit in a water bath set at 35 °C. First, thionyl chloride (150 mmol) was added to a three neck round bottom flask. Second, while slightly shaking the flask, 2-decenoic acid (100 mmol) was added. Once both compounds were in the flask, the flask was connected to a condenser system, sealed, and reacted for five hours. After reaction completion, the synthesized 2-decenoyl chloride was removed from the flask and stored until later use. Decanoyl chloride (DC) and hexanoyl chloride (HC) were purchased from Sigma Fisher (USA).

### 4.4. Acylation Reactions

The direct acylation of chitosan materials by acyl chlorides was achieved by first making a 5 mg mL^−1^ solution of chitosan material in pyridine. With a ratio of 3:1 (*v*/*v*) pyridine to acyl chloride, the acyl chloride was slowly added while stirring. The solution reacted for 1.5 h. Once the reaction was complete, the chitosan materials were removed and placed in 10% acetone solution (1 L), then removed and placed in 70% ethanol solution, removed and finally placed in deionized water (DI). Each step lasted for at least one hour. After the final washing step, the chitosan materials were removed from the solution, placed flat onto a glass surface, and frozen at −80 °C. The frozen materials were lyophilized. After lyophilization, the materials were stored in a desiccator until further analysis.

### 4.5. Scanning Electron Microscopy

Images were acquired using SEM (Nova NANOSEM 650 FEI™, Hillsboro, OR, USA) to determine the effects of acylation on fiber size and surface morphology. Twenty fibers were randomly selected in each image of as-spun and treated membranes and fiber diameter was measured using ImageJ.

### 4.6. FTIR

Attenuated total reflectance (ATR) Fourier transform infrared (FTIR) spectra were collected with a diamond crystal using an FTIR spectrometer (Frontier, Perkin-Elmer, Waltham, MA, USA). ATR spectra were collected to confirm the attachment of FA groups to the chitosan polymer chain and TFA salt removal by the treatments.

### 4.7. Thermogravimetric Analysis

Thermogravimetry analysis (TGA) was performed with a TGA-Q50 (TA Instruments, New Castle, DE, USA) under a nitrogen atmosphere. The heating rate was 10 °C/min.

### 4.8. Contact Angle

Water contact angles of modified membranes were determined using a VCA optima measurement machine (AST products, INC, Billerica, MA, USA) [14]. Water droplets (5 μL) were placed carefully onto the membrane surfaces. A digital camera recorded the photographs of the droplets after approximately one minute. The goniometry software of VCA OptimaXE calculated the contact angles. For each modification, four different membranes were tested at three regions.

### 4.9. Antimicrobial Activity

*Pseudomonas aeruginosa* (*P. aeruginosa,* ATCC #27317) and *Staphylococcus aureus* (*S aureus*, UAMS-1, a clinical osteomyelitis strain) grown overnight were diluted to 1:50 and 1:10, respectively. Diluted bacteria (500 μL) were added to the well containing HC, DC, 2DC modified membranes, sponge, or gauze, and incubated for 24 h. The membranes, sponges, and gauzes were taken out of the solution after the incubation period and washed three times with 500 μL of 1× phosphate-buffered saline (PBS). They were then immersed in 500 μL of sterilized tryptic soy broth (TSB) and sonicated for 5 min to detach the bacteria. After sonication, the detached bacteria solution was used for colony forming unit (CFU) counting by plating dilutions.

### 4.10. Cytocompatibility

NIH 3T3 (American Type Culture Collection, RRID:CVCL_0594) fibroblasts were seeded at a concentration of 10^4^ cells cm^−2^ in a 24-well plate in Dulbecco’s Modified Eagle’s Medium (DMEM) high glucose supplemented with 10% fetal bovine serum (FBS, Gibco) and 2% (100 µg mL^−1^) Normocin (InvivoGen, San Diego, CA, USA). Chitosan membranes were placed into well inserts and then immersed into the wells containing cells and media. Control wells did not have any materials added (tissue culture plastic). Plates were incubated at 37 °C with 5% carbon dioxide (CO_2_). Every 24 h, the inserts were removed, the wells were bright field imaged, and the media was refreshed. After 48 h, viability was determined using CellTiter-Glo^®^ (Promega, Madison, WI, USA) and expressed as a percentage of tissue culture plastic controls.

### 4.11. Statistical Analysis

SigmaPlot and GraphPad Prism 7.2 software (GraphPad Software Incorporation, La Jolla, CA, USA) was used to perform the statistical analysis. Data were assessed first by performing Shapiro–Wilk normality test, followed by Brown–Forsythe equal variance test. If both passed, a one-way analysis of variance (ANOVA) further analyzed the data, followed by Holm–Sidak post hoc analysis to detect significance between experimental groups (α = 0.05). Kruskal–Wallis ANOVA on ranks, followed by Tukey post hoc test, completed additional analysis if necessary normality and equal variance requirements did not occur.

## Figures and Tables

**Table 1:** Summary of TGA analysis for HC, DC, and 2DC modified chitosan membranes, and chitosan (86.5% DDA).

Sample	Weight Loss (%)			Weight Loss w/o Water (%)		T_D_ (I) (°C)	T_D_ (Onset) (°C)
	Water	Deg-1	Remaining	Deg-1 (Norm)	Remaining (Norm)	Deg-1 Initiation	Deg-1 Onset
	rt–150 °C	150–500 °C	at 500 °C	150–500 °C	at 500 °C		
Chitosan	11	49	40	55	45	263	295
HC	4	76	20	79	21	215	280
DC	8	69	23	75	25	217	262
2DC	3	77	20	79	21	212	263

## Data Availability

The data presented in this study are available on request from the corresponding author.
